# Supplementary material for: The Genetic Architecture of Emerging Fungicide Resistance in Populations of a Global Wheat Pathogen
Source: Genome Biol Evol. 2020 Sep 28;12(12):2231–44. doi: 10.1093/gbe/evaa203 (PMC7846115; doi:10.1093/gbe/evaa203)
Supplement: evaa203_Supplementary_Data [file evaa203_supplementary_data.pdf]

Supplementary Information

The genetic architecture of emerging fungicide resistance in populations of a global wheat pathogen

Supplementary Figures

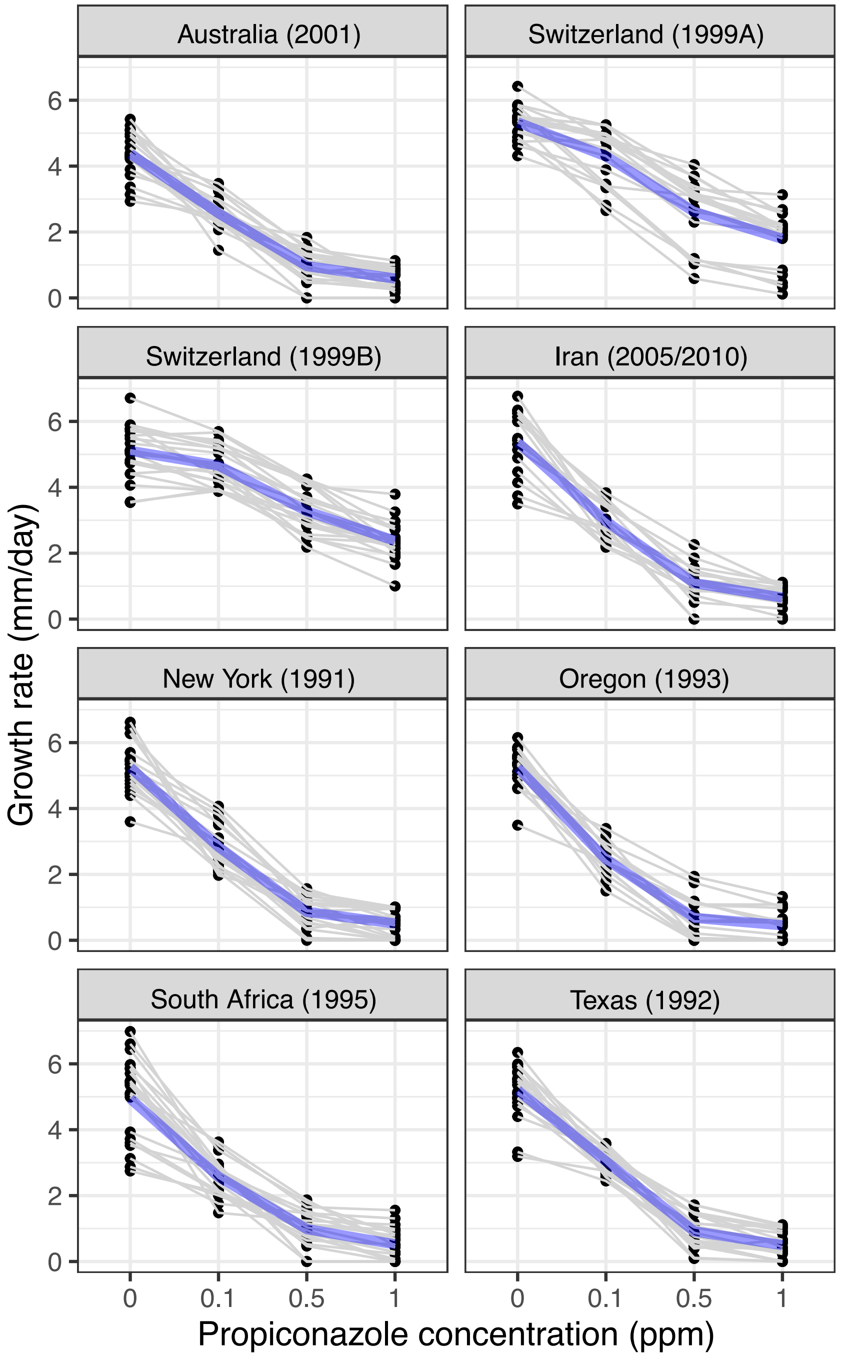

Supplementary Figure 1: Growth rate measures reflecting the reduction in colony development for increasing fungicide concentrations. Grey lines represent individual isolates. Blue lines show the population average.

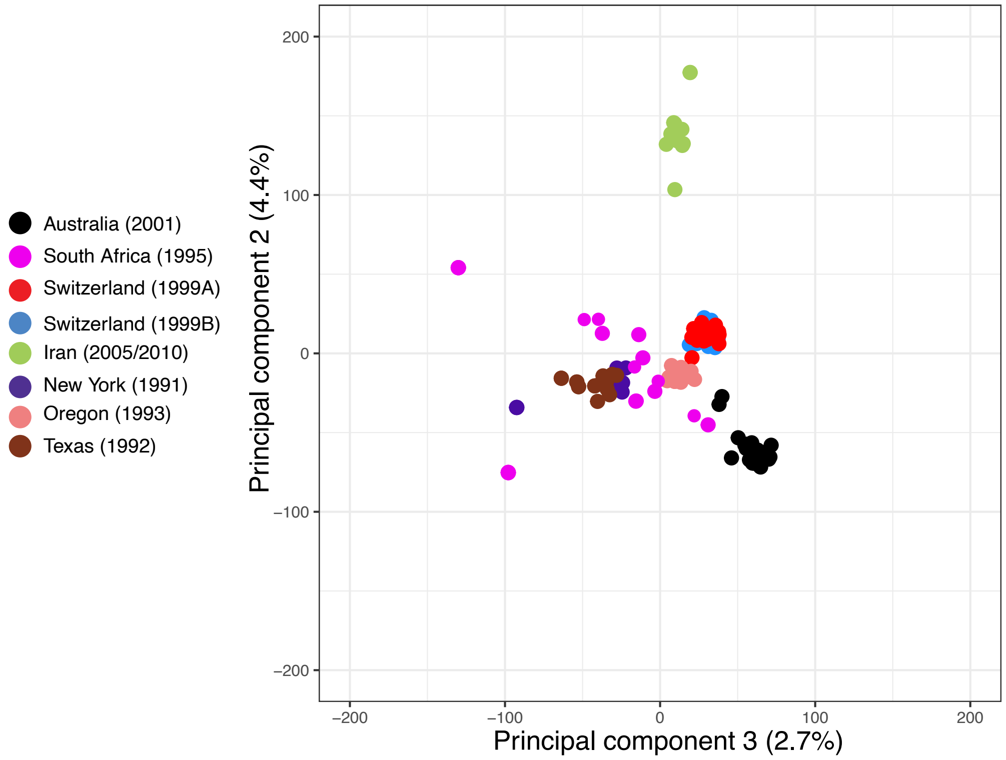

Supplementary Figure 2: Second and third principal components from a PCA of 436,365 genome-wide SNPs. Populations are color-coded

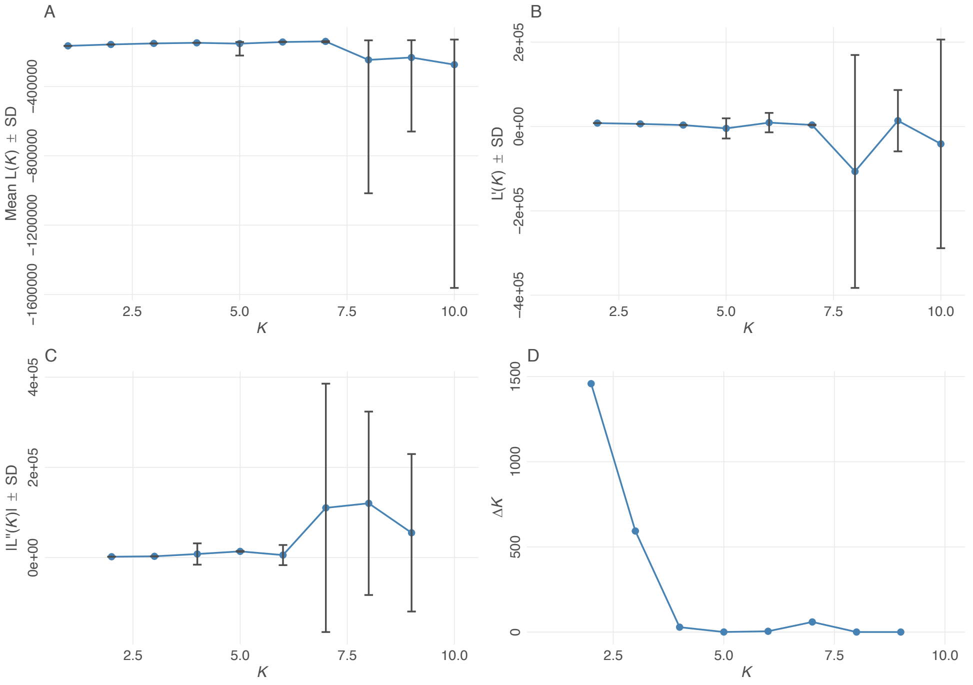

Supplementary Figure 3: Evaluation of the cluster assignments using STRUCTURE. The panels show the mean estimated *ln* probability of the data, based on the first derivative per K and delta K. (A) Mean likelihood and variance per *K* value. (B) The rate of change of the likelihood distribution. (C) The absolute value of the second order rate of change of the likelihood distribution. (D) Mean delta *K* plot from K=2 to 9.

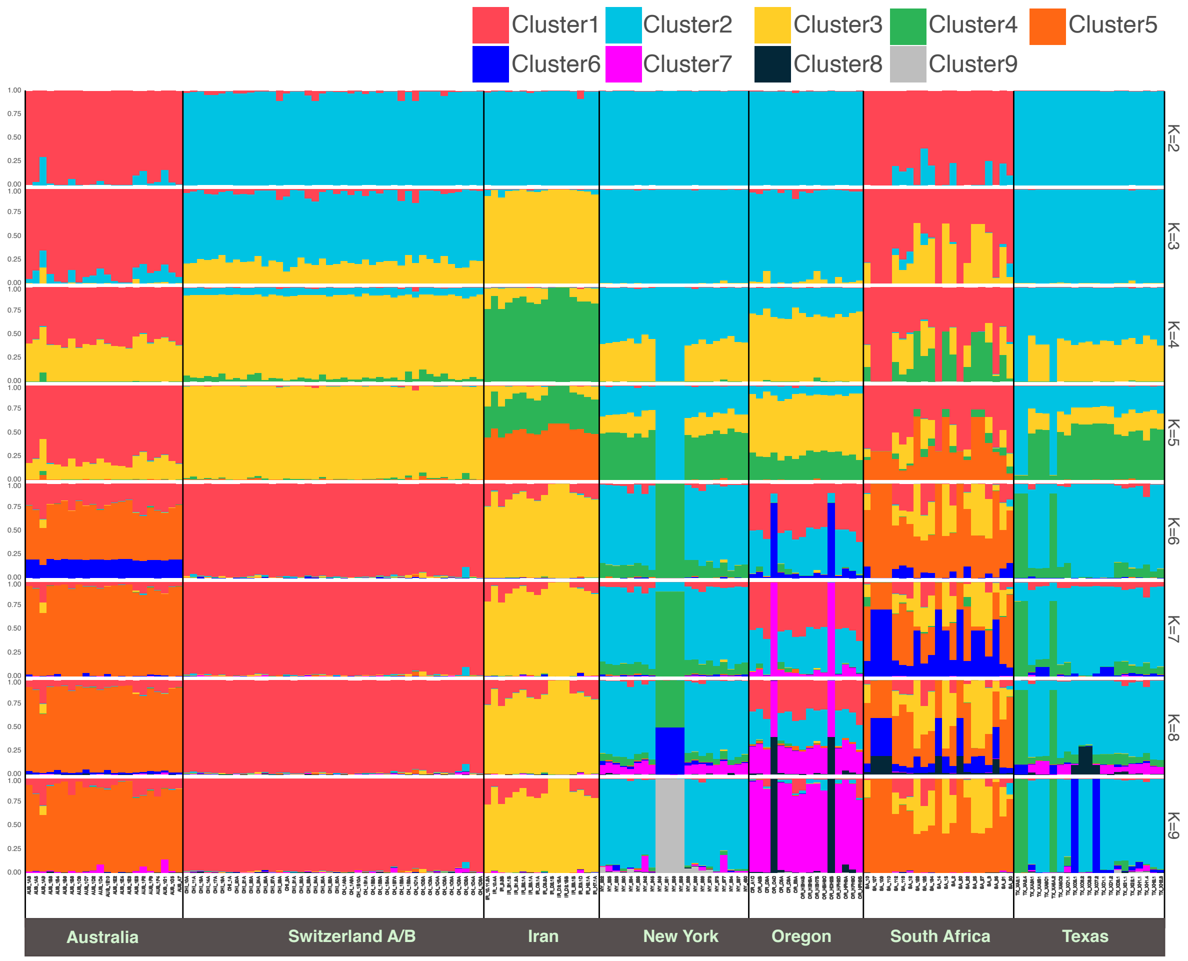

Supplementary Figure 4. Bayesian genetic clustering of 159 isolates of *Parastagonospora nodorum* based on 2348 single nucleotide polymorphic markers using STRUCTURE. Vertical colored bars represent the assignment probability of each isolate for different values of K.

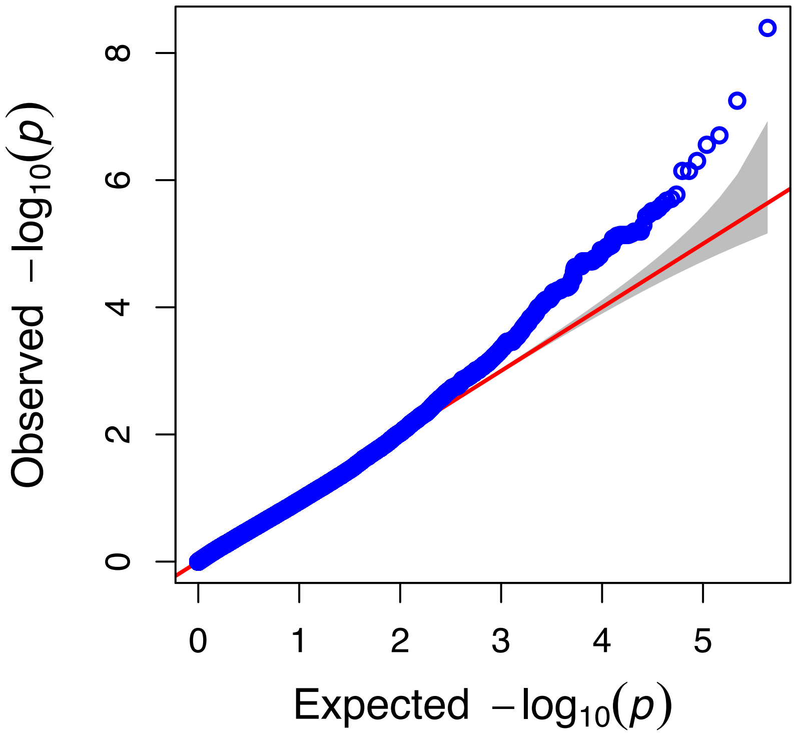

Supplementary Figure 5: Quantile-Quantile (QQ) plots. The -$\log_{10}$ *P*-values obtained for the genome wide association using EC_{50} scores (blue dots). The red continuous line indicates the expected values and the grey interval shows the confidence interval.

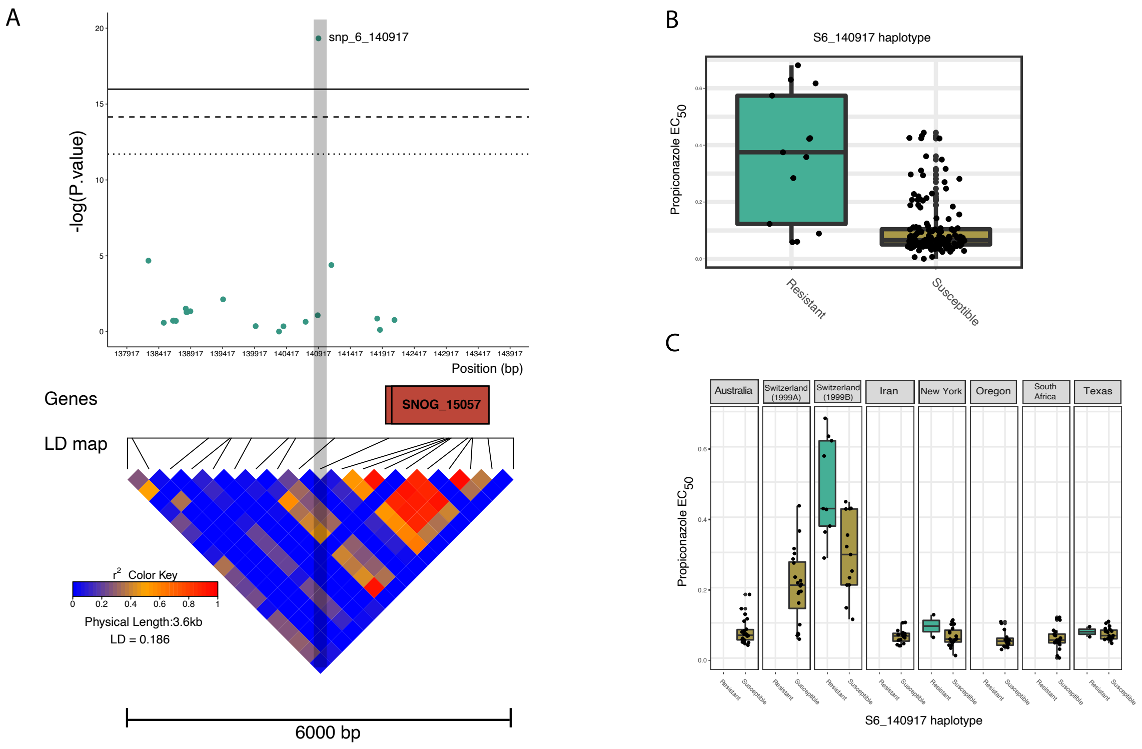

Supplementary Figure 6: **Detailed analysis of markers associated with fungicide resistance in *Parastagonospora nodorum*.** (A) *P*-value distribution around snp_6_140917. In genes is shown a rectangle representing the SNOG_15057 gene, which encodes a helix-loop-helix (HLH) domain. The heatmap of pairwise linkage disequilibrium r^2 for SNPs considering all 159 isolates. The region spans a total of 6 kbp. (B) Boxplots of EC_{50} values for the group of isolates possessing the resistant versus the susceptible allele at snp_6_140917. (C) Boxplots of EC_{50} values between isolates carrying the resistant versus the susceptible allele organized according to population.

```
Sn2000.fa      1 MDSTGRSRSCSQSQPRRSDNGPLHPILPSSFTDDHSVYHHGDRERTHVDADATSFHTTETERRILESSSGSSSDDDVDEKLEAEEEKYKV
AUS_1F4.fa     1 MDSIGRSRSCSQSQPRRSDNGPLHPILPSSFTDDHSVYHHGDRERTHVDADATSFHTTETERRILESSSGSSSDDDVDEKLEAEEEKYKV
AUS_1G1.fa     1 MDSTGRSRSCSQSQPRRSDNGPLHPILPSSFTDDHSVYHHGDRERTHVDADATSFHTTETERRILESSSGSSSDDDVDEKLEAEEEKYKV
CH_1B1A.fa     1 MDSIGRSRSCSQSQPRRSDNGPLHPILPSSFTDDHSVYHHGDRERTHVDADATSFHTTETERRILESSSGSSSDDDVDEKLEAEEEKYKV
CH_1B2A.fa     1 MDSTGRSRSCSQSQPRRSDNGPLHPILPSSFTDDHSVYHHGDRERTHVDADATSFHTTETERRILESSSGSSSDDDVDEKLEAEEEKYKV
IR_E3.1B.fa    1 MDSTGRSRSCSQSQPRRSDNGPLHPILPSSFTDDHSVYHHGDRERTHVDADATSFHTTETERRILESSSGSSSDDDVDEKLEAEEEKYKV
IR_E3.1D.fa    1 MDSIGRSRSCSQSQPRRSDNGPLHPILPSSFTDDHSVYHHGDRERTHVDADATSFHTTETERRILESSSGSSSDDDVDEKLEAEEEKYKV
OR_HBH4B.fa    1 MDSTGRSRSCSQSQPRRSDNGPLHPILPSSFTDDHSVYHHGDRERTHVDADATSFHTTETERRILESSSGSSSDDDVDEKLEAEEEKYKV
OR_HBH5A.fa    1 MDSIGRSRSCSQSQPRRSDNGPLHPILPSSFTDDHSVYHHGDRERTHVDADATSFHTTETERRILESSSGSSSDDDVDEKLEAEEEKYKV
TX_XG1.1.fa    1 MDSTGRSRSCSQSQPRRSDNGPLHPILPSSFTDDHSVYHHGDRERTHVDADATSFHTTETERRILESSSGSSSDDDVDEKLEAEEEKYKV
TX_XG2.1.fa    1 MDSIGRSRSCSQSQPRRSDNGPLHPILPSSFTDDHSVYHHGDRERTHVDADATSFHTTETERRILESSSGSSSDDDVDEKLEAEEEKYKV
CHI_13A.fa     1 MDSTGRSRSCSQSQPRRSDNGPLHPILPSSFTDDHSVYHHGDRERTHVDADATSFHTTETERRILESSSGSSSDDDVDEKLEAEEEKYKV
CHI_19A.fa     1 MDSIGRSRSCSQSQPRRSDNGPLHPILPSSFTDDHSVYHHGDRERTHVDADATSFHTTETERRILESSSGSSSDDDVDEKLEAEEEKYKV

Sn2000.fa     91 AHEQKTTYEEKRGSVPYEHDVEAQPAALEKKGMSVSIKDANLVTWDSDDDPYNPKNWSTKRKWAVTLFVSCFTVGAPISSSMIAPALSSI
AUS_1F4.fa    91 AHEQKTTYEEKRGSVPYEHDVEAQPAALEKKGMSVSIKDANLVTWDSDDDPYNPKNWSTKRKWAVTLFVSCFTVGSPISSSMIAPALSSI
AUS_1G1.fa    91 AHEQKTTYEEKRGSVPYEHDVEAQPAALEKKGMSVSIKDANLVTWDSDDDPYNPKNWSTKRKWAVTLFVSCFTVGAPISSSMIAPALSSI
CH_1B1A.fa    91 AHEQKTTYEEKRGSVPYEHDVEAQPAALEKKGMSVSIKDANLVTWDSDDDPYNPKNWSTKRKWAVTLFVSCFTVGSPISSSMIAPALSSI
CH_1B2A.fa    91 AHEQKTTYEEKRGSVPYEHDVEAQPAALEKKGMSVSIKDANLVTWDSDDDPYNPKNWSTKRKWAVTLFVSCFTVGAPISSSMIAPALSSI
IR_E3.1B.fa   91 AHEQKTTYEEKRGSVPYEHDVEAQPAALEKKGMSVSIKDANLVTWDSDDDPYNPKNWSTKRKWAVTLFVSCFTVGAPISSSMIAPALSSI
IR_E3.1D.fa   91 AHEQKTTYEEKRGSVPYEHDVEAQPAALEKKGMSVSIKDANLVTWDSDDDPYNPKNWSTKRKWAVTLFVSCFTVGSPISSSMIAPALSSI
OR_HBH4B.fa   91 AHEQKTTYEEKRGSVPYEHDVEAQPAALEKKGMSVSIKDANLVTWDSDDDPYNPKNWSTKRKWAVTLFVSCFTVGAPISSSMIAPALSSI
OR_HBH5A.fa   91 AHEQKTTYEEKRGSVPYEHDVEAQPAALEKKGMSVSIKDANLVTWDSDDDPYNPKNWSTKRKWAVTLFVSCFTVGSPISSSMIAPALSSI
TX_XG1.1.fa   91 AHEQKTTYEEKRGSVPYEHDVEAQPAALEKKGMSVSIKDANLVTWDSDDDPYNPKNWSTKRKWAVTLFVSCFTVGAPISSSMIAPALSSI
TX_XG2.1.fa   91 AHEQKTTYEEKRGSVPYEHDVEAQPAALEKKGMSVSIKDANLVTWDSDDDPYNPKNWSTKRKWAVTLFVSCFTVGSPISSSMIAPALSSI
CHI_13A.fa    91 AHEQKTTYEEKRGSVPYEHDVEAQPAALEKKGMSVSIKDANLVTWDSDDDPYNPKNWSTKRKWAVTLFVSCFTVGAPISSSMIAPALSSI
CHI_19A.fa    91 AHEQKTTYEEKRGSVPYEHDVEAQPAALEKKGMSVSIKDANLVTWDSDDDPYNPKNWSTKRKWAVTLFVSCFTVGSPISSSMIAPALSSI

Sn2000.fa    181 SAEFGMTKGVMSQIPLSIFVLGYAVGPLFLGPLSEIYGRVIILQLSNLFYLLNNLGCGFAQTSGQLMAFRFLSGLGGSAPLAISGGVISD
AUS_1F4.fa   181 SAEFGMTKGVMSQIPLSIFVLGYAVGPLFLGPLSEIYGRVIILQLSNLFYLLNNLGCGFAQTSGQLMAFRFLSGLGGSAPLAISGGVISD
AUS_1G1.fa   181 SAEFGMTKGVMSQIPLSIFVLGYAVGPLFLGPLSEIYGRVIILQLSNLFYLLNNLGCGFAQTSGQLMAFRFLSGLGGSAPLAISGGVISD
CH_1B1A.fa   181 SAEFGMTKGVMSQIPLSIFVLGYAVGPLFLGPLSEIYGRVIILQLSNLFYLLNNLGCGFAQTSGQLMAFRFLSGLGGSAPLAISGGVISD
CH_1B2A.fa   181 SAEFGMTKGVMSQIPLSIFVLGYAVGPLFLGPLSEIYGRVIILQLSNLFYLLNNLGCGFAQTSGQLMAFRFLSGLGGSAPLAISGGVISD
IR_E3.1B.fa  181 SAEFGMTKGVMSQIPLSIFVLGYAVGPLFLGPLSEIYGRVIILQLSNLFYLLNNLGCGFAQTSGQLMAFRFLSGLGGSAPLAISGGVISD
IR_E3.1D.fa  181 SAEFGMTKGVMSQIPLSIFVLGYAVGPLFLGPLSEIYGRVIILQLSNLFYLLNNLGCGFAQTSGQLMAFRFLSGLGGSAPLAISGGVISD
OR_HBH4B.fa  181 SAEFGMTKGVMSQIPLSIFVLGYAVGPLFLGPLSEIYGRVIILQLSNLFYLLNNLGCGFAQTSGQLMAFRFLSGLGGSAPLAISGGVISD
OR_HBH5A.fa  181 SAEFGMTKGVMSQIPLSIFVLGYAVGPLFLGPLSEIYGRVIILQLSNLFYLLNNLGCGFAQTSGQLMAFRFLSGLGGSAPLAISGGVISD
TX_XG1.1.fa  181 SAEFGMTKGVMSQIPLSIFVLGYAVGPLFLGPLSEIYGRVIILQLSNLFYLLNNLGCGFAQTSGQLMAFRFLSGLGGSAPLAISGGVISD
TX_XG2.1.fa  181 SAEFGMTKGVMSQIPLSIFVLGYAVGPLFLGPLSEIYGRVIILQLSNLFYLLNNLGCGFAQTSGQLMAFRFLSGLGGSAPLAISGGVISD
CHI_13A.fa   181 SAEFGMTKGVMSQIPLSIFVLGYAVGPLFLGPLSEIYGRVIILQLSNLFYLLNNLGCGFAQTSGQLMAFRFLSGLGGSAPLAISGGVISD
CHI_19A.fa   181 SAEFGMTKGVMSQIPLSIFVLGYAVGPLFLGPLSEIYGRVIILQLSNLFYLLNNLGCGFAQTSGQLMAFRFLSGLGGSAPLAISGGVISD

Sn2000.fa    271 TFFPEQRGRAISIYSLAPLLGPAIGPILGGFISQDTTWRWCFYATTIFSAVMQCFSLFFLEETYAPKLLQWKRDKLSEETGNRELYTEYD
AUS_1F4.fa   271 TFFPEQRGRAISIYSLAPLLGPAIGPILGGFISQDTTWRWCFYATTIFSAVMQCFSLFFLEETYAPKLLQWKRDKLSEETGNRELYTEYD
AUS_1G1.fa   271 TFFPEQRGRAISIYSLAPLLGPAIGPILGGFISQDTTWRWCFYATTIFSAVMQCFSLFFLEETYAPKLLQWKRDKLSEETGNRELYTEYD
CH_1B1A.fa   271 TFFPEQRGRAISIYSLAPLLGPAIGPILGGFISQDTTWRWCFYATTIFSAVMQCFSLFFLEETYAPKLLQWKRDKLSEETGNRELYTEYD
CH_1B2A.fa   271 TFFPEQRGRAISIYSLAPLLGPAIGPILGGFISQDTTWRWCFYATTIFSAVMQCFSLFFLEETYAPKLLQWKRDKLSEETGNRELYTEYD
IR_E3.1B.fa  271 TFFPEQRGRAISIYSLAPLLGPAIGPILGGFISQDTTWRWCFYATTIFSAVMQCFSLFFLEETYAPKLLQWKRDKLSEETGNRELYTEYD
IR_E3.1D.fa  271 TFFPEQRGRAISIYSLAPLLGPAIGPILGGFISQDTTWRWCFYATTIFSAVMQCFSLFFLEETYAPKLLQWKRDKLSEETGNRELYTEYD
OR_HBH4B.fa  271 TFFPEQRGRAISIYSLAPLLGPAIGPILGGFISQDTTWRWCFYATTIFSAVMQCFSLFFLEETYAPKLLQWKRDKLSEETGNRELYTEYD
OR_HBH5A.fa  271 TFFPEQRGRAISIYSLAPLLGPAIGPILGGFISQDTTWRWCFYATTIFSAVMQCFSLFFLEETYAPKLLQWKRDKLSEETGNRELYTEYD
TX_XG1.1.fa  271 TFFPEQRGRAISIYSLAPLLGPAIGPILGGFISQDTTWRWCFYATTIFSAVMQCFSLFFLEETYAPKLLQWKRDKLSEETGNRELYTEYD
TX_XG2.1.fa  271 TFFPEQRGRAISIYSLAPLLGPAIGPILGGFISQDTTWRWCFYATTIFSAVMQCFSLFFLEETYAPKLLQWKRDKLSEETGNRELYTEYD
CHI_13A.fa   271 TFFPEQRGRAISIYSLAPLLGPAIGPILGGFISQDTTWRWCFYATTIFSAVMQCFSLFFLEETYAPKLLQWKRDKLSEETGNRELYTEYD
CHI_19A.fa   271 TFFPEQRGRAISIYSLAPLLGPAIGPILGGFISQDTTWRWCFYATTIFSAVMQCFSLFFLEETYAPKLLQWKRDKLSEETGNRELYTEYD

Sn2000.fa    361 DADRTLLLTLKMAIKRPFKLLFTQPIVQVLACYMAFIYGLMYLMLSTFPLLWTDRYGQSASMGSLNYISMAVGFFLGVQISAPFNDMLYR
AUS_1F4.fa   361 DADRTLLLTLKMAIKRPFKLLFTQPIVQVLACYMAFIYGLMYLMLSTFPLLWTDRYGQSASMGSLNYISMAVGFFLGVQISAPFNDMLYR
AUS_1G1.fa   361 DADRTLLLTLKMAIKRPFKLLFTQPIVQVLACYMAFIYGLMYLMLSTFPLLWTDRYGQSASMGSLNYISMAVGFFLGVQISAPFNDMLYR
CH_1B1A.fa   361 DADRTLLLTLKMAIKRPFKLLFTQPIVQVLACYMAFIYGLMYLMLSTFPLLWTDRYGQSASMGSLNYISMAVGFFLGVQISAPFNDMLYR
CH_1B2A.fa   361 DADRTLLLTLKMAIKRPFKLLFTQPIVQVLACYMAFIYGLMYLMLSTFPLLWTDRYGQSASMGSLNYISMAVGFFLGVQISAPFNDMLYR
IR_E3.1B.fa  361 DADRTLLLTLKMAIKRPFKLLFTQPIVQVLACYMAFIYGLMYLMLSTFPLLWTDRYGQSASMGSLNYISMAVGFFLGVQISAPFNDMLYR
IR_E3.1D.fa  361 DADRTLLLTLKMAIKRPFKLLFTQPIVQVLACYMAFIYGLMYLMLSTFPLLWTDRYGQSASMGSLNYISMAVGFFLGVQISAPFNDMLYR
OR_HBH4B.fa  361 DADRTLLLTLKMAIKRPFKLLFTQPIVQVLACYMAFIYGLMYLMLSTFPLLWTDRYGQSASMGSLNYISMAVGFFLGVQISAPFNDMLYR
OR_HBH5A.fa  361 DADRTLLLTLKMAIKRPFKLLFTQPIVQVLACYMAFIYGLMYLMLSTFPLLWTDRYGQSASMGSLNYISMAVGFFLGVQISAPFNDMLYR
TX_XG1.1.fa  361 DADRTLLLTLKMAIKRPFKLLFTQPIVQVLACYMAFIYGLMYLMLSTFPLLWTDRYGQSASMGSLNYISMAVGFFLGVQISAPFNDMLYR
TX_XG2.1.fa  361 DADRTLLLTLKMAIKRPFKLLFTQPIVQVLACYMAFIYGLMYLMLSTFPLLWTDRYGQSASMGSLNYISMAVGFFLGVQISAPFNDMLYR
CHI_13A.fa   361 DADRTLLLTLKMAIKRPFKLLFTQPIVQVLACYMAFIYGLMYLMLSTFPLLWTDRYGQSASMGSLNYISMAVGFFLGVQISAPFNDMLYR
CHI_19A.fa   361 DADRTLLLTLKMAIKRPFKLLFTQPIVQVLACYMAFIYGLMYLMLSTFPLLWTDRYGQSASMGSLNYISMAVGFFLGVQISAPFNDMLYR

Sn2000.fa    451 RLKKKNNGAGKPEFRIPIMVPASLLVPIGLFWYGWSAQAKLHWIMPNIGACIFCAGTIVSFQCIQTYLVDSYTRYAASATAAAIFLRSLA
AUS_1F4.fa   451 RLKKKNNGAGKPEFRIPIMVPASLLVPIGLFWYGWSAQAKLHWIMPNIGACIFCAGTIVSFQCIQTYLVDSYTRYAASATAAAIFLRSLA
AUS_1G1.fa   451 RLKKKNNGAGKPEFRIPIMVPASLLVPIGLFWYGWSAQAKLHWIMPNIGACIFCAGTIVSFQCIQTYLVDSYTRYAASATAAAIFLRSLA
CH_1B1A.fa   451 RLKKKNNGAGKPEFRIPIMVPASLLVPIGLFWYGWSAQAKLHWIMPNIGACIFCAGTIVSFQCIQTYLVDSYTRYAASATAAAIFLRSLA
CH_1B2A.fa   451 RLKKKNNGAGKPEFRIPIMVPASLLVPIGLFWYGWSAQAKLHWIMPNIGACIFCAGTIVSFQCIQTYLVDSYTRYAASATAAAIFLRSLA
IR_E3.1B.fa  451 RLKKKNNGAGKPEFRIPIMVPASLLVPIGLFWYGWSAQAKLHWIMPNIGACIFCAGTIVSFQCIQTYLVDSYTRYAASATAAAIFLRSLA
IR_E3.1D.fa  451 RLKKKNNGVGKPEFRIPIMVPASLLVPIGLFWYGWSAQAKLHWIMPNIGACIFCAGTIVSFQCIQTYLVDSYTRYAASATAAAIFLRSLA
OR_HBH4B.fa  451 RLKKKNNGAGKPEFRIPIMVPASLLVPIGLFWYGWSAQAKLHWIMPNIGACIFCAGTIVSFQCIQTYLVDSYTRYAASATAAAIFLRSLA
OR_HBH5A.fa  451 RLKKKNNGVGKPEFRIPIMVPASLLVPIGLFWYGWSAQAKLHWIMPNIGACIFCAGTIVSFQCIQTYLVDSYTRYAASATAAAIFLRSLA
TX_XG1.1.fa  451 RLKKKNNGAGKPEFRIPIMVPASLLVPIGLFWYGWSAQAKLHWIMPNIGACIFCAGTIVSFQCIQTYLVDSYTRYAASATAAAIFLRSLA
TX_XG2.1.fa  451 RLKKKNNGAGKPEFRIPIMVPASLLVPIGLFWYGWSAQAKLHWIMPNIGACIFCAGTIVSFQCIQTYLVDSYTRYAASATAAAIFLRSLA
CHI_13A.fa   451 RLKKKNNGAGKPEFRIPIMVPASLLVPIGLFWYGWSAQAKLHWIMPNIGACIFCAGTIVSFQCIQTYLVDSYTRYAASATAAAIFLRSLA
CHI_19A.fa   451 RLKKKNNGAGKPEFRIPIMVPASLLVPIGLFWYGWSAQAKLHWIMPNIGACIFCAGTIVSFQCIQTYLVDSYTRYAASATAAAIFLRSLA

Sn2000.fa    541 GFGFPLFAPAMYNALDFGWGNSLLGFVALSLGVPAPFLLWKFGETLRERSKFAAG
AUS_1F4.fa   541 GFGFPLFAPAMYNALDFGWGNSLLGFVALSLGVPAPFLLWKFGETLRERSKFAAG
AUS_1G1.fa   541 GFGFPLFAPAMYNALDFGWGNSLLGFVALSLGVPAPFLLWKFGETLRERSKFAAG
CH_1B1A.fa   541 GFGFPLFAPAMYNALDFGWGNSLLGFVALSLGVPAPFLLWKFGETLRERSKFAAG
CH_1B2A.fa   541 GFGFPLFAPAMYNALDFGWGNSLLGFVALSLGVPAPFLLWKFGETLRERSKFAAG
IR_E3.1B.fa  541 GFGFPLFAPAMYNALDFGWGNSLLGFVALSLGVPAPFLLWKFGETLRERSKFAAG
IR_E3.1D.fa  541 GFGFPLFAPAMYNALDFGWGNSLLGFVALSLGVPAPFLLWKFGETLRERSKFAAG
OR_HBH4B.fa  541 GFGFPLFAPAMYNALDFGWGNSLLGFVALSLGVPAPFLLWKFGETLRERSKFAAG
OR_HBH5A.fa  541 GFGFPLFAPAMYNALDFGWGNSLLGFVALSLGVPAPFLLWKFGETLRERSKFAAG
TX_XG1.1.fa  541 GFGFPLFAPAMYNALDFGWGNSLLGFVALSLGVPAPFLLWKFGETLRERSKFAAG
TX_XG2.1.fa  541 GFGFPLFAPAMYNALDFGWGNSLLGFVALSLGVPAPFLLWKFGETLRERSKFAAG
CHI_13A.fa   541 GFGFPLFAPAMYNALDFGWGNSLLGFVALSLGVPAPFLLWKFGETLRERSKFAAG
CHI_19A.fa   541 GFGFPLFAPAMYNALDFGWGNSLLGFVALSLGVPAPFLLWKFGETLRERSKFAAG
```

Supplementary Figure 7: Multiple sequence alignment for the SNOG_14185 amino acid sequence showing isolates from the global distribution. The black triangle in the amino acid position four indicate the non-synonymous change identified in the GWAS. Isolates harboring an isoleucine (I) were found to be associated to higher EC50 values compared to isolates containing a threonine (T).

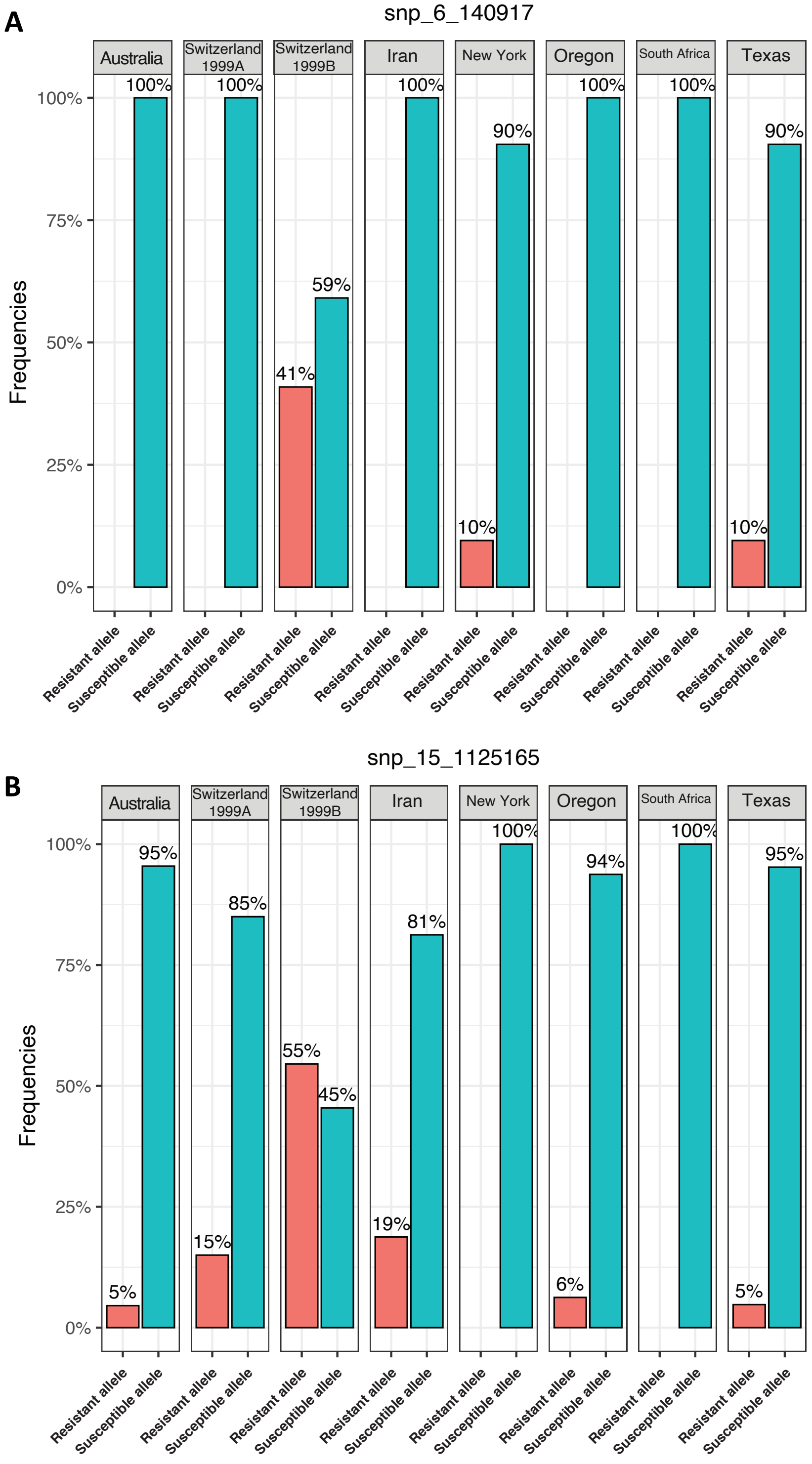

Supplementary Figure 8: Frequencies of resistant and susceptible alleles within populations of *Parastagonospora nodorum*. (*A*) SNP marker snp_6_140917 and (*B*) snp_15_1125165.

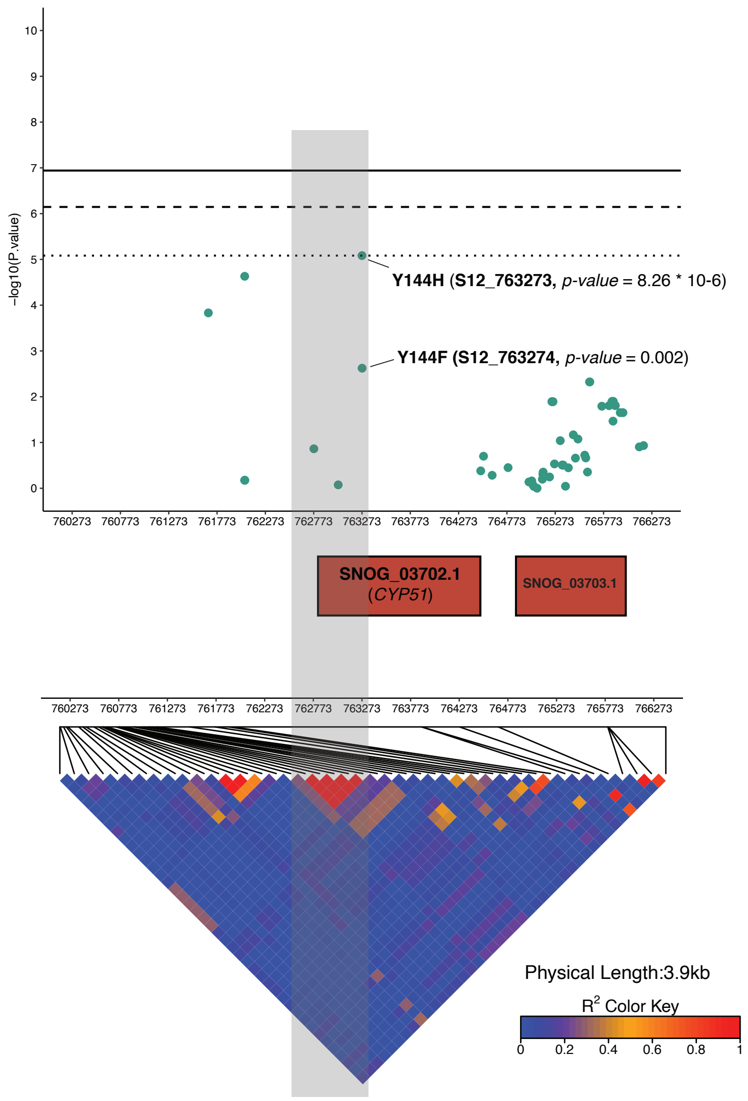

Supplementary Figure 9: **Detailed analysis of SNP markers associated with fungicide resistance in the *CYP51* gene.** *P*-value distribution around SNOG_03702.1 (*CYP51*). SNPs underlying non-synonymous substitutions at amino acid position 144 are highlighted. Horizontal lines indicate significance thresholds for false discovery rate (FDR) 10% (dotted line), FDR 5% (dashed line) and Bonferroni correction (solid line). The heatmap shows pairwise linkage disequilibrium r^2 for SNPs considering all 159 isolates. The region spans a total of 6 kb.

Supplementary Tables

Supplementary Table 1. Raw growth rate data from individual isolates of *Parastagonospora nodorum* (see also Pereira *et al.* Fungal Genetics and Biology 2020).

isolate	population	mean	dose	mean	dose	mean	dose	mean	dose
AUS_1A3	AUS	4.26042319	0	2.24910828	0.1	0.87559366	0.5	0.2414014	
AUS_1A5	AUS	3.73073124	0	2.99201898	0.1	1.34337241	0.5	0.99650089	1
AUS_1A9	AUS	5.2345575	0	1.4454414	0.1	0	0.5	0	1
AUS_1B3	AUS	2.9323024	0	2.52684146	0.1	0.48051343	0.5	0.69239035	1
AUS_1B4	AUS	4.90379598	0	2.51644551	0.1	0.59517335	0.5	0.38134226	1
AUS_1B6	AUS	4.37775396	0	2.93858644	0.1	0.46271305	0.5	0.37685659	1
AUS_1B8	AUS	4.22026211	0	2.06836486	0.1	0.81263908	0.5	0.69389127	1
AUS_1C3	AUS	4.31387855	0	3.24974436	0.1	1.12812813	0.5	0.8872045	1
AUS_1C7	AUS	3.90052016	0	2.52980424	0.1	1.34456607	0.5	0.45292742	1
AUS_1D2	AUS	4.9764659	0	2.39593943	0.1	0.49926211	0.5	0.25653751	1
AUS_1D4	AUS	5.42221287	0	2.3847329	0.1	0	0.5	0	1
AUS_1E10	AUS	5.11036884	0	2.70881335	0.1	1.13284266	0.5	0.75971553	1
AUS_1E2	AUS	3.36559331	0	2.38782481	0.1	1.83538398	0.5	0.16260693	1
AUS_1E4	AUS	4.32773036	0	3.47353305	0.1	1.48826372	0.5	1.13299743	1
AUS_1E6	AUS	5.07852859	0	3.28202381	0.1	1.55941056	0.5	0.83121755	1
AUS_1E8	AUS	4.73999368	0	2.5590608	0.1	0.76172413	0.5	0.27937632	1
AUS_1F2	AUS	4.31893737	0	2.71562018	0.1	1.2839678	0.5	0.90254822	1
AUS_1F3	AUS	4.30620291	0	2.63929102	0.1	1.13472771	0.5	0.82271936	1
AUS_1F4	AUS	4.40349063	0	2.4084717	0.1	0.95462988	0.5	0.4269348	1
AUS_1G1	AUS	3.14041442	0	2.32482345	0.1	1.26585069	0.5	0.72986646	1
AUS_1G5	AUS	4.5588213	0	2.47655618	0.1	0.74411795	0.5	1.01476239	1
AUS_A7	AUS	4.54711164	0	2.20675343	0.1	1.51841218	0.5	0.88205028	1
CH_1A3A	CH_A	4.87518341	0	2.81164132	0.1	1.1953733	0.5	0.35020024	1
CH_1A9A	CH_A	5.85783211	0	4.74255574	0.1	3.39383577	0.5	2.56764129	1
CH_1B10A	CH_A	5.68848523	0	3.34455033	0.1	1.12882347	0.5	0.84490753	1
CH_1B1A	CH_A	5.44940654	0	4.83517872	0.1	4.0465681	0.5	2.56820895	1
CH_1B2A	CH_A	5.82188993	0	3.45269197	0.1	1.15581654	0.5	0.70024366	1
CH_1B3A	CH_A	5.02369812	0	4.30891071	0.1	2.29871265	0.5	2.01126355	1
CH_1B4A	CH_A	4.31442535	0	3.39088239	0.1	3.10009596	0.5	2.22848046	1
CH_1B7A	CH_A	5.38989749	0	2.65143123	0.1	0.5866352	0.5	0.11545133	1
CH_1B8A	CH_A	5.47299448	0	5.25583509	0.1	3.67392393	0.5	2.12956417	1
CH_1B9A	CH_A	5.49947556	0	5.24153389	0.1	3.06750914	0.5	1.89189495	1
CH_1C1A	CH_A	5.50907082	0	4.83700202	0.1	3.2138977	0.5	1.91571226	1
CH_1C2A	CH_A	5.49852584	0	4.98490756	0.1	3.30253275	0.5	3.13258337	1
CH_1C3A	CH_A	5.0530118	0	4.53574158	0.1	2.73156244	0.5	1.89802829	1
CH_1C4A	CH_A	4.61523572	0	3.88557861	0.1	2.61444734	0.5	2.19294834	1

CH_1C5A	CH_A	6.42068184	0	4.78494078	0.1	3.69872808	0.5	2.11935142	1
CH_1C6A	CH_A	5.33772679	0	4.93692545	0.1	3.08032759	0.5	2.15873271	1
CH_1C9A	CH_A	5.32074102	0	4.52533725	0.1	3.00542461	0.5	2.06295025	1
CH_1D2A	CH_A	5.85649461	0	5.23169364	0.1	3.14252305	0.5	2.67957906	1
CH_1D4A	CH_A	4.7413707	0	4.80387423	0.1	2.72459721	0.5	1.80145516	1
CH_1D6A	CH_A	4.7804996	0	3.41153129	0.1	1.03457761	0.5	0.45418657	1
CHI_10A	CH_B	5.89404002	0	4.28789785	0.1	3.28793635	0.5	2.48590143	1
CHI_11A	CH_B	4.40633375	0	3.87599727	0.1	3.10507625	0.5	2.14599119	1
CHI_13A	CH_B	5.12778503	0	4.61445821	0.1	2.82457853	0.5	2.18015209	1
CHI_14A	CH_B	5.02536059	0	4.7339339	0.1	3.71194775	0.5	2.72532621	1
CHI_17A	CH_B	3.55995806	0	3.89452352	0.1	2.5632087	0.5	2.43913137	1
CHI_19A	CH_B	6.70815078	0	5.69326123	0.1	4.22795133	0.5	2.24783571	1
CHI_1A	CH_B	5.47794461	0	5.19783986	0.1	4.2526762	0.5	2.72657788	1
CHI_20A	CH_B	4.75531084	0	4.4386559	0.1	3.52442494	0.5	2.43641424	1
CHI_21A	CH_B	4.06291567	0	3.88687873	0.1	2.89367606	0.5	2.39317901	1
CHI_22A	CH_B	5.11411342	0	4.68262581	0.1	2.83686364	0.5	2.37514859	1
CHI_24A	CH_B	5.76975815	0	5.16429331	0.1	4.16248129	0.5	2.01874072	1
CHI_25A	CH_B	4.41616794	0	4.58259088	0.1	2.4550209	0.5	1.9462104	1
CHI_27A	CH_B	5.33308364	0	5.04257741	0.1	3.62402811	0.5	2.97188123	1
CHI_28A	CH_B	5.56582165	0	4.48768984	0.1	2.1815958	0.5	1.00508468	1
CHI_2A	CH_B	5.07545606	0	3.88806324	0.1	3.27359667	0.5	2.13957569	1
CHI_31A	CH_B	5.70849272	0	5.43261045	0.1	3.59182505	0.5	2.81005549	1
CHI_32A	CH_B	4.81958042	0	4.18454974	0.1	3.01297336	0.5	1.88319503	1
CHI_33A	CH_B	5.57055893	0	5.66521732	0.1	4.04607901	0.5	3.79246759	1
CHI_34A	CH_B	5.89336941	0	5.27237863	0.1	3.27543664	0.5	2.31450131	1
CHI_35A	CH_B	3.53902736	0	3.94840735	0.1	2.49309453	0.5	1.66101845	1
CHI_52A	CH_B	4.73635525	0	4.1836506	0.1	2.45434528	0.5	2.31668267	1
CHI_60A	CH_B	5.78298596	0	5.36706873	0.1	4.24566445	0.5	3.25368225	1
IR_10.11.2A	IR	4.89006221	0	2.21020416	0.1	1.14498698	0.5	0.85335872	1
IR_10.4A	IR	5.48536988	0	3.43958771	0.1	0.51001487	0.5	0.31690405	1
IR_9.3B	IR	4.14413429	0	2.45121027	0.1	1.04893892	0.5	0.57856111	1
IR_B1.1B	IR	5.42222261	0	3.03379256	0.1	0.92121274	0.5	0.50488349	1
IR_B1.2A	IR	6.76503158	0	2.78221003	0.1	0	0.5	0	1
IR_B2.1A	IR	5.13334813	0	3.41127422	0.1	1.85242351	0.5	0.98638842	1
IR_B3.1A	IR	6.24763166	0	2.61163188	0.1	0.71826858	0.5	0.06639537	1
IR_C3.1A	IR	3.74075178	0	2.76149723	0.1	1.34884304	0.5	1.05226754	1
IR_C5.2A	IR	6.00554404	0	2.37848173	0.1	1.12837415	0.5	0.59626148	1
IR_D2.1B	IR	6.31521372	0	2.95878939	0.1	0	0.5	0	1
IR_D2.1BA	IR	5.99959362	0	3.83450915	0.1	2.26241036	0.5	1.01906322	1
IR_D2.1BB	IR	6.34234121	0	3.53873087	0.1	1.46205068	0.5	0.88074419	1
IR_E3.1B	IR	4.47017793	0	2.18024719	0.1	0.84763091	0.5	0.81507499	1
IR_E3.1D	IR	3.49539167	0	2.71719826	0.1	1.56804579	0.5	1.11594222	1
IR_H2.1A	IR	5.30776199	0	3.51429939	0.1	1.48550615	0.5	0.65805755	1
IR_H7.1A	IR	6.13344304	0	3.67077353	0.1	1.18246918	0.5	0.95407764	1

NY_322	NY	6.44645882	0	1.97218464	0.1	1.24717648	0.5	0.99251781	1
NY_325	NY	4.95595298	0	2.62766921	0.1	0.33556008	0.5	0	1
NY_328	NY	4.74758343	0	2.7024889	0.1	0.49441509	0.5	0.33614332	1
NY_330	NY	5.21810617	0	3.79846938	0.1	1.14238607	0.5	1.01256082	1
NY_334	NY	5.49269015	0	2.078449	0.1	0	0.5	0	1
NY_335	NY	4.51301355	0	2.96183981	0.1	0.91459804	0.5	0.52715445	1
NY_342	NY	5.34661924	0	2.54021688	0.1	0.55737672	0.5	0	1
NY_345	NY	5.03559338	0	2.45664224	0.1	0.73502883	0.5	0.71386673	1
NY_349	NY	6.27576748	0	3.50273759	0.1	1.35137298	0.5	0.99191496	1
NY_351	NY	5.0398772	0	3.10978465	0.1	1.4543245	0.5	1.00854507	1
NY_356	NY	5.69852782	0	4.06924053	0.1	1.56703652	0.5	0.70729571	1
NY_358	NY	5.43973832	0	3.92795662	0.1	0.91387364	0.5	0.05256818	1
NY_363	NY	4.85059372	0	2.23310457	0.1	0.88423665	0.5	0.95514675	1
NY_366	NY	3.59871735	0	2.78650319	0.1	0.89589774	0.5	0.45384296	1
NY_369	NY	5.44193831	0	2.68607997	0.1	1.0892264	0.5	0.95329074	1
NY_373	NY	6.61902659	0	2.29387369	0.1	0.06858579	0.5	0	1
NY_375	NY	6.27465282	0	2.55232984	0.1	0.68142658	0.5	0	1
NY_377	NY	5.0601119	0	2.52599607	0.1	1.43458865	0.5	0.60944984	1
NY_384	NY	5.06269967	0	3.56835843	0.1	1.20932156	0.5	0.10267333	1
NY_387	NY	4.63611636	0	2.9853907	0.1	0.36239897	0.5	0.6467709	1
NY_430	NY	4.39547888	0	2.14109654	0.1	0.75991163	0.5	0.60487107	1
OR_A1C	OR	6.15271178	0	2.77469699	0.1	1.09066309	0.5	0.97941163	1
OR_A2B	OR	5.78885246	0	2.20336017	0.1	0	0.5	0	1
OR_C3A	OR	5.38804994	0	2.42466536	0.1	0.42366146	0.5	0.15973326	1
OR_C4D	OR	4.92014326	0	1.49832763	0.1	0	0.5	0	1
OR_C5A	OR	4.60375708	0	3.39434521	0.1	1.94011649	0.5	1.32925089	1
OR_C8A	OR	5.60765458	0	3.17133686	0.1	0.06845238	0.5	0	1
OR_E3A	OR	3.49353009	0	2.37729986	0.1	1.13498861	0.5	0.57639586	1
OR_HBH4B	OR	5.53311565	0	2.40862512	0.1	0.57231594	0.5	0.65612197	1
OR_HBH5A	OR	5.52363462	0	2.60117795	0.1	1.18727524	0.5	0.43150236	1
OR_HBH7B	OR	5.85749577	0	2.37027088	0.1	1.08643437	0.5	1.08675741	1
OR_HBH8C	OR	5.35033784	0	1.78585902	0.1	0	0.5	0	1
OR_HDH2B	OR	5.0131035	0	2.94437325	0.1	1.74759848	0.5	1.01685162	1
OR_HFH2D	OR	5.56126079	0	2.8558818	0.1	0.57846466	0.5	0.53818538	1
OR_HFH3A	OR	5.31174721	0	2.34669902	0.1	0.79415366	0.5	0.50581956	1
OR_HFH6C	OR	5.12000485	0	1.98003444	0.1	0.20867341	0.5	0	1
OR_HFH9B	OR	4.62583442	0	2.17507105	0.1	0.06549095	0.5	0	1
SA_10	SA	2.87383764	0	2.00309963	0.1	0.95017727	0.5	0	1
SA_107	SA	5.97586898	0	2.85260037	0.1	0	0.5	0	1
SA_109	SA	5.11040256	0	3.6286585	0.1	1.86974355	0.5	0.48772799	1
SA_110	SA	5.70625245	0	2.78759219	0.1	0.46792626	0.5	0	1
SA_112	SA	6.43588184	0	2.95350348	0.1	0	0.5	0	1
SA_116	SA	5.84601407	0	1.47807343	0.1	1.23661293	0.5	1.11745754	1
SA_12	SA	3.52392143	0	2.76937932	0.1	0.84811382	0.5	0.28872199	1

SA_123	SA	6.98044955	0	2.63686772	0.1	0.78850947	0.5	0.01652514	1
SA_125	SA	5.46221025	0	2.78825183	0.1	1.12965704	0.5	0.94317975	1
SA_134	SA	5.87118705	0	3.52913025	0.1	1.65288067	0.5	1.55552362	1
SA_14	SA	6.60409572	0	3.37943317	0.1	1.79021715	0.5	0.8316371	1
SA_15	SA	3.12551874	0	1.75513497	0.1	1.46513168	0.5	1.30677925	1
SA_2	SA	2.74603437	0	2.0953173	0.1	0.64084271	0.5	0.52003764	1
SA_20	SA	4.99053513	0	2.70727842	0.1	1.54209549	0.5	0.68742526	1
SA_22	SA	5.06711569	0	2.15625458	0.1	0.74160336	0.5	0.0754809	1
SA_25	SA	5.48059468	0	2.55217307	0.1	1.44208548	0.5	0.98392575	1
SA_27	SA	3.71733363	0	2.13555878	0.1	0	0.5	0	1
SA_3	SA	5.34917556	0	2.35831164	0.1	0.52443412	0.5	0.22927987	1
SA_35	SA	3.93084302	0	2.81016278	0.1	1.37585277	0.5	0.65295934	1
SA_61	SA	5.41056988	0	2.54267605	0.1	1.03877589	0.5	0.43057726	1
SA_80	SA	3.60506701	0	2.08671964	0.1	1.14683929	0.5	0.7847365	1
TX_XA2.1	TX	5.52170028	0	2.77747401	0.1	0.75802193	0.5	0.23035831	1
TX_XA2.4	TX	5.72385267	0	2.58297663	0.1	0.0912528	0.5	0	1
TX_XA3A1	TX	5.90702745	0	3.56620557	0.1	0.46544769	0.5	0.35895348	1
TX_XA3B1	TX	4.71729191	0	3.16043698	0.1	0.87172174	0.5	0.29259311	1
TX_XA3C1	TX	5.99278549	0	3.11438566	0.1	0.48081211	0.5	0	1
TX_XA5A.2	TX	5.35713335	0	2.66657045	0.1	1.03719102	0.5	0.55268481	1
TX_XA5C2	TX	5.14413915	0	2.99679852	0.1	0.49561362	0.5	0.0170093	1
TX_XC1.1	TX	5.98277502	0	2.55854369	0.1	0.63757088	0.5	0.31871937	1
TX_XC5.1	TX	4.9758677	0	3.25948135	0.1	1.71625957	0.5	1.06638914	1
TX_XC6.2	TX	4.95333291	0	3.1665546	0.1	0.11136029	0.5	0	1
TX_XC6.3	TX	4.94506789	0	3.21342108	0.1	1.46990587	0.5	0.65336332	1
TX_XC7.2	TX	5.55387386	0	3.42189334	0.1	1.4433055	0.5	0.43317051	1
TX_XD1.1	TX	5.44634599	0	3.19712517	0.1	1.71683252	0.5	1.11729209	1
TX_XD1.2	TX	5.76355046	0	2.99486583	0.1	0.39600828	0.5	0.6620424	1
TX_XD3.1	TX	5.09169013	0	3.40352516	0.1	1.16885002	0.5	0.85850801	1
TX_XG1.1	TX	4.82378145	0	2.70038976	0.1	1.13980272	0.5	1.05321183	1
TX_XG2.1	TX	4.40020523	0	3.36491653	0.1	1.4504304	0.5	0.92551145	1
TX_XH1.1	TX	5.91051649	0	3.58286895	0.1	1.06595326	0.5	0.59297552	1
TX_XH1.4	TX	3.32452788	0	2.44387021	0.1	0.59127846	0.5	0.27614561	1
TX_XH2.1	TX	6.34543131	0	3.00804126	0.1	0.596852	0.5	0	1
TX_XH2.3	TX	3.18825185	0	2.74788685	0.1	1.48723189	0.5	1.01675225	1

Supplementary Table 2. Number of principal components (PCs) included in the GWAS, the Bayesian Information Criteria (BIC) value and the likelihood values.

Number of PCs/Covariates	BIC (larger is better) - Schwarz 1978	log Likelihood Function Value
0	155.23	162.83
1	153.04	163.18
2	150.60	163.28
3	150.50	165.70

Supplementary Table 3. The top loci from the genome-wide association mapping in *Parastagonospora nodorum* for different significance thresholds. Coordinates and distances are given in base pairs.

Threshold	p-value	SNP	Chromosome	Position	P.value	maf	Start_gene	End_gene	gene ID	distance
Bonferonni	1.16E-07	S6_140917	6	140917	4.03E-09	0.08	141961	143580	SNOG_15057	-1044
Bonferonni	1.16E-07	S15_1125165	15	1125165	5.62E-08	0.14	1123292	1125175	SNOG_14185	0
FDR_5%	7.15E-07	S12_743935	12	743935	1.97E-07	0.19	742669	743805	SNOG_03692	130
FDR_5%	7.15E-07	S15_1003265	15	1003265	2.77E-07	0.08	1001315	1002569	SNOG_14240	696
FDR_5%	7.15E-07	S22_739749	22	739749	4.99E-07	0.12	739857	741023	SNOG_10460	-108
FDR_5%	7.15E-07	S22_739131	22	739131	7.15E-07	0.09	738155	738916	SNOG_10462	215
FDR_5%	7.15E-07	S22_739134	22	739134	7.15E-07	0.09	738155	738916	SNOG_10462	218
FDR_10%	8.26E-06	S7_999491	7	999491	1.69E-06	0.16	999693	1002904	SNOG_06551	-202
FDR_10%	8.26E-06	S15_995131	15	995131	1.97E-06	0.12	993566	994895	SNOG_14244	236
FDR_10%	8.26E-06	S10_1112832	10	1112832	2.10E-06	0.06	1111907	1112980	SNOG_05992	0
FDR_10%	8.26E-06	S2_2489215	2	2489215	2.42E-06	0.06	2486059	2486877	SNOG_30129	2338
FDR_10%	8.26E-06	S7_1000371	7	1000371	2.78E-06	0.14	999693	1002904	SNOG_06551	0
FDR_10%	8.26E-06	S7_999855	7	999855	3.07E-06	0.14	999693	1002904	SNOG_06551	0
FDR_10%	8.26E-06	S7_999973	7	999973	3.07E-06	0.14	999693	1002904	SNOG_06551	0
FDR_10%	8.26E-06	S15_1124326	15	1124326	3.45E-06	0.12	1123292	1125175	SNOG_14185	0
FDR_10%	8.26E-06	S4_690420	4	690420	3.72E-06	0.24	688597	689699	SNOG_30188	721
FDR_10%	8.26E-06	S15_997120	15	997120	5.12E-06	0.13	995750	996587	SNOG_14243	533
FDR_10%	8.26E-06	S2_2115190	2	2115190	6.43E-06	0.06	2113412	2114494	SNOG_02160	696
FDR_10%	8.26E-06	S2_2115259	2	2115259	6.43E-06	0.06	2116921	2118744	SNOG_02157	-1662
FDR_10%	8.26E-06	S7_1000797	7	1000797	6.44E-06	0.12	999693	1002904	SNOG_06551	0
FDR_10%	8.26E-06	S7_999559	7	999559	6.44E-06	0.12	999693	1002904	SNOG_06551	-134
FDR_10%	8.26E-06	S9_1398308	9	1398308	6.86E-06	0.07	1398685	1405487	SNOG_08614	-377
FDR_10%	8.26E-06	S8_1059071	8	1059071	7.07E-06	0.06	1059052	1059496	SNOG_07538	0
FDR_10%	8.26E-06	S7_999266	7	999266	7.28E-06	0.12	998455	999165	SNOG_30471	101
FDR_10%	8.26E-06	S7_999349	7	999349	7.28E-06	0.12	998455	999165	SNOG_30471	184
FDR_10%	8.26E-06	S7_999820	7	999820	7.28E-06	0.12	999693	1002904	SNOG_06551	0
FDR_10%	8.26E-06	S7_999823	7	999823	7.28E-06	0.12	999693	1002904	SNOG_06551	0
FDR_10%	8.26E-06	S7_999307	7	999307	7.28E-06	0.12	998455	999165	SNOG_30471	142

FDR_10%	8.26E-06	S7_999816	7	999816	7.28E-06	0.12	999693	1002904	SNOG_06551	0
FDR_10%	8.26E-06	S20_60249	20	60249	7.42E-06	0.18	61041	63647	SNOG_12292	-792
FDR_10%	8.26E-06	S8_1057002	8	1057002	7.50E-06	0.06	1054678	1057518	SNOG_07536	0
FDR_10%	8.26E-06	S20_60214	20	60214	8.21E-06	0.14	56340	58034	SNOG_12290	2180
FDR_10%	8.26E-06	S20_763661	20	763661	8.26E-06	0.11	767389	768302	SNOG_13193	-3728
FDR_10%	8.26E-06	S12_763273	12	763273	8.26E-06	0.16	762821	764490	SNOG_03702	0
